# Impact of the COVID-19 Pandemic on Mental Health and Quality of Life among Local Residents in Liaoning Province, China: A Cross-Sectional Study

**DOI:** 10.3390/ijerph17072381

**Published:** 2020-03-31

**Authors:** Yingfei Zhang, Zheng Feei Ma

**Affiliations:** 1 Mathematics Teaching and Research Office, Public Basic College, Jinzhou Medical University, Jinzhou 121001, China; 2 Department of Health and Environmental Sciences, Xi’an Jiaotong-Liverpool University, Suzhou 215123, China; Zhengfeei.Ma@xjtlu.edu.cn

**Keywords:** coronavirus, mental health, IES, pandemic, China

## Abstract

Our study aimed to investigate the immediate impact of the COVID-19 pandemic on mental health and quality of life among local Chinese residents aged ≥18 years in Liaoning Province, mainland China. An online survey was distributed through a social media platform between January and February 2020. Participants completed a modified validated questionnaire that assessed the Impact of Event Scale (IES), indicators of negative mental health impacts, social and family support, and mental health-related lifestyle changes. A total of 263 participants (106 males and 157 females) completed the study. The mean age of the participants was 37.7 ± 14.0 years, and 74.9% had a high level of education. The mean IES score in the participants was 13.6 ± 7.7, reflecting a mild stressful impact. Only 7.6% of participants had an IES score ≥26. The majority of participants (53.3%) did not feel helpless due to the pandemic. On the other hand, 52.1% of participants felt horrified and apprehensive due to the pandemic. Additionally, the majority of participants (57.8–77.9%) received increased support from friends and family members, increased shared feeling and caring with family members and others. In conclusion, the COVID-19 pandemic was associated with mild stressful impact in our sample, even though the COVID-19 pandemic is still ongoing. These findings would need to be verified in larger population studies.

## 1. Introduction

Since December 2019, there has been an outbreak of pneumonia of an unknown etiology that was first reported in Wuhan, Hubei Province, China [1]. Following the outbreak, a novel coronavirus, SARS-CoV-2, was identified as the causative virus for the pandemic in China and other parts of the world by the World Health Organization (WHO) [2]. By 12 February 2020, there were 43,103 confirmed cases of COVID-19, and of these, 42,708 cases (99.1%) were from China [3]. As these data indicate, China has been severely affected by the COVID-19, which has been a major public health disaster [4].

COVID-19 has been considered a relative of severe acute respiratory syndrome (SARS), which has the possibility of transmission from animals to humans [5]. Currently, it is still unclear when the pandemic will reach its peak. To date, the source of the SARS-CoV-2 remains unknown. However, the SARS-CoV-2 infection has been associated with contact with a local seafood vendor in Wuhan that illegally sold some wildlife animals including bats [6].

During the COVID-19 pandemic, it is imperative to understand how the population, especially those in the severely affected countries such as China, have been coping with such a major disaster. The devastation caused by COVID-19 may be comparable to that caused by the SARS epidemic in 2003. The SARS epidemic caused >8000 infections and 800 deaths worldwide (in 26 countries) [7,8]. The SARS epidemic was controlled within eight months (by July 2003) [2]. Moderate-to-severe post-traumatic stress symptoms were also reported among the population in areas severely affected by the SARS epidemic [9]. Risk factors such as being female were associated with a higher risk of developing SARS-related post-traumatic stress symptoms [9]. Similarly, the impacts of MERS, H1N1 and Ebola epidemics on mental health including depression and substance use disorders have also been recorded [8]. Additionally, the populations may have experienced some known risk factors for depression and anxiety including high mortality rate, resource and food insecurity, discrimination, and experience with infected and sick individuals, which can lead to some adverse mental health outcomes during these epidemics [8]. In contrast, there have been >82,000 cases of COVID-19 with >2800 deaths within two months since the outbreak began in December 2019 [2]. To date, there are limited studies that have investigated how severe the impact of COVID-19 pandemic is on mental health and quality of life.

Some recent studies published in the Lancet have reported the clinical symptoms of patients infected with COVID-19 and forecasted the spread of COVID-19 [8,10,11]. However, few studies have reported the impact of the COVID-19 pandemic on mental health or quality of life in mainland China, even though the pandemic has severely affected China and many other parts of the world. Therefore, the study aimed to investigate whether there was an immediate impact of the COVID-19 pandemic on mental health, its related lifestyle habits and quality of life among Chinese adults in mainland China one week after Wuhan was locked down and travel restrictions were imposed by the Chinese government.

## 2. Materials and Methods

A cross-sectional study was performed from 28 January 2020 until 5 February 2020. Only adults (aged ≥18 years) of Chinese nationality who were able to provide verbal informed consent were recruited in the study using convenience and snowball sampling methods. Additionally, they were required to be living in Jinzhou, Liaoning Province, mainland China throughout the pandemic period including pre-COVID-19 and COVID-19 periods. The pre-COVID-19 and COVID-19 periods were defined as the period from November 2019 until December 2019, and from January 2020 until February 2020, respectively. To ensure that participants were still living in the city, they were asked to provide the name of the city they resided during these periods in the questionnaire. No monetary rewards were given for completing the questionnaire. The study protocol was approved by the Ethics Committee of Jinzhou Medical University (ref. no. JYDLL2020002).

### 2.1. Impact of Event Scale (IES)

Participants were asked to complete an online socio-demographic questionnaire (e.g., sex, age and self-reported BMI) via WeChat and phone interviews. Participant personal information including names was anonymized to maintain and protect confidentiality. Additionally, a modified validated Chinese version of a 15-item IES with a Cronbach’s alpha of 0.84 was used to assess the extent of traumatic stress (excessive panic and anxiety) including trauma-related distressing memories and persistent negative emotions resulting from the pandemic [12]. The response for each question was scored 0 (not at all), 1 (rarely), 3 (sometimes) or 5 (often), with a lower score indicating a less stressful impact. There were two subscales in the questionnaire, namely, the intrusive and avoidance subscales. A cut-off of the IES ≥26 was used to reflect moderate-to-severe impact.

### 2.2. Other Indicators of Negative Mental Health Impact

Participants were also asked to complete six modified and validated questions regarding negative mental health impacts before and resulting from the pandemic; these questions had a Cronbach’s alpha of 0.88 [12]. These following domains were assessed changes in stress from work, financial stress, stress from home, horrified feelings due to the COVID-19 pandemic, apprehensive due to the COVID-19 pandemic, and helpless feelings due to the COVID-19 pandemic (response options for each: much decreased, decreased, unchanged/same as before, increased, and much increased).

### 2.3. Impact on Social and Family Support

Participants also completed a modified and validated questionnaire investigating the impact of the COVID-19 pandemic on social and family support (Cronbach’s alpha of 0.87). The five questions in this questionnaire evaluated support from friends, support from family members, sharing feelings with other family members, sharing feelings with others, and caring for family members’ feelings [12]. The response options for these questions were as follows: much decreased, decreased, unchanged/same as before, increased, and much increased. A lower score indicated lower social and family support [12].

### 2.4. Impact on Mental Health-Related Lifestyle Changes

Using a modified and validated questionnaire investigating the impact of the COVID-19 pandemic on mental health-related lifestyle changes, participants were asked to rate whether they were paying less or more attention to their mental health before and during the outbreak (much decreased, decreased, unchanged/same as before, increased, and much increased) [12]. Additionally, participants were asked to indicate whether they were spending less or more time to rest, relax and exercise before and during the outbreak (response options: much decreased, decreased, unchanged/same as before, increased, and much increased). Participant responses to these four questions were calculated using the Mental Health Lifestyle Scale (MHLSS) (Cronbach’s alpha value of 0.82) [12]. A lower MHLSS score was used to indicate less favorable changes in lifestyle [12].

### 2.5. Statistical Analysis

Statistical analysis was performed using SPSS ver. 16.0 (IBM, Chicago, IL, USA). All results of quantitative variables were reported either as mean ± standard deviation or frequency (percentage) (%). A Chi-square test was employed to assess if there was a significant association between categorical variables. An unpaired–test was used to determine whether there was a difference in the IES scores between categorical variables between genders. General linear model (GLM) multivariate analysis was employed to assess the difference in dependent variables and independent variables including age groups. A *P*-value < 0.05 was considered to be statistically significant.

## 3. Results

### 3.1. Participant Characteristics

Of the 400 participants who were invited, 263 participants (i.e., 106 males and 157 females) were recruited into the study with a response rate of 65.8% (Table 1). Those who declined the study invitation (*n* = 137) provided reasons as follows: no time to complete the questionnaire (60.6%), currently not living in Jinzhou (*n* = 9.5%), and not interested (*n* = 29.9%%). The mean BMI of participants was 22.9 kg/m^2^, indicating normal BMI. The mean age of participants was 37.7 ± 14.0, and 41.4% were aged between 18 and 30 years. More than half of the participants (74.9%) had a higher level of education. Additionally, 60.8% of participants were married at the time of the study. In terms of employment status, 52.5% of participants had a full-time job, 31.6 were students (31.6%), and 16.0% had a part-time job. The majority of participants (95.1%) reported no religion; a minority followed Buddhism (4.2%) and Christianity (0.8%).

### 3.2. IES

The overall mean IES score in participants was 13.6 ± 7.7, reflecting a mild stressful impact (Table 2). There was no difference in mean IES scores between females and males (14.2 vs. 12.8, respectively) (*P* = 0.173). Overall, only 7.6% of participants had an IES score ≥26. There was no association between the percentages of participants with an IES ≥26 and sex (male: 6.4%; female: 9.4%, respectively) (*P* = 0.478). Other sociodemographic variables including age group and education level were not associated with the IES score or the percentage of participants with an IES ≥26. Additionally, none of these variables significantly predicted the IES score in the multiple regression analysis (Table 2). The overall mean scores for the intrusion and avoidance scales in participants were 12.7 ± 2.6 and 13.4 ± 2.9, respectively. Only the mean intrusive score in males was significantly higher than that in females (13.0 vs. 12.3) (*P* = 0.027). There was no association between the mean intrusive scale score and other demographic factors (*P* > 0.05). There was also no association between the mean avoidance scale score and any demographic factors (*P* > 0.05).

### 3.3. Other Indicators of Negative Mental Health Impact

Following the onset of the pandemic, more than half of the participants (69.2%) reported no increased stress from work (Table 3). Additionally, 76.8% mentioned that they did not experience increased financial stress arising from the pandemic. A total of 74.5% of participants reported that they did not experience increased stress from home.

On the other hand, 52.1% of participants reported that they felt horrified and apprehensive due to the COVID-19 pandemic. However, the majority of participants (53.3%) did not feel helpless due to the pandemic. There was a significant association between different age groups and some of the responses including “feel horrified due to the COVID-19 pandemic” (*P* = 0.002); “feel apprehensive due to the COVID-19 pandemic” (*P* = 0.001); and “feel helpless due to the COVID-19 pandemic” (*P* = 0.049). Other sociodemographic variables including sex and education level were not associated with the indicators of negative mental health impact.

### 3.4. Impact on Social and Family Support

Following the onset of the pandemic, the majority of participants reported that they received increased support from friends (64.6%) and increased support from family members (63.9%). The majority also experienced and increased shared feelings with family members (57.8%), increased shared feelings with others when feeling blue (62.4%), and increased caring for family members’ feelings (77.9%) (Table 4).

Overall, participants aged between 41–50 years were less likely to have experienced increased support from friends, increased support from family members, increased shared feelings with family members, increased shared feelings with others when they felt blue, and increased caring for family members’ feelings than those in other age groups (all *P* < 0.05) (Table 4). Other sociodemographic variables, including age and education levels, were not significantly associated with the items in the questionnaires.

### 3.5. Impact on Mental Health-Related Lifestyle Changes

There were 67.7% of participants who reported that they were paying more attention to their mental health following the pandemic (Table 5). Additionally, 62.0% of participants reported that they were spending more time to rest. The majority of participants (64.2%) stated that they were spending more time to relax. More than half of the participants (59.7%) also reported that they were spending more time exercising.

Age group was associated with lifestyle changes among participants. Participants aged 18–30, 31–40 and >50 years were reported to have spent significantly more time relaxing. Participants who spent more time resting were more likely to have a lower IES score (*P* = 0.028). Other sociodemographic variables, including age and education levels were not significantly associated with questions regarding mental health-related lifestyle changes. 

## 4. Discussion

**Figure 1:**
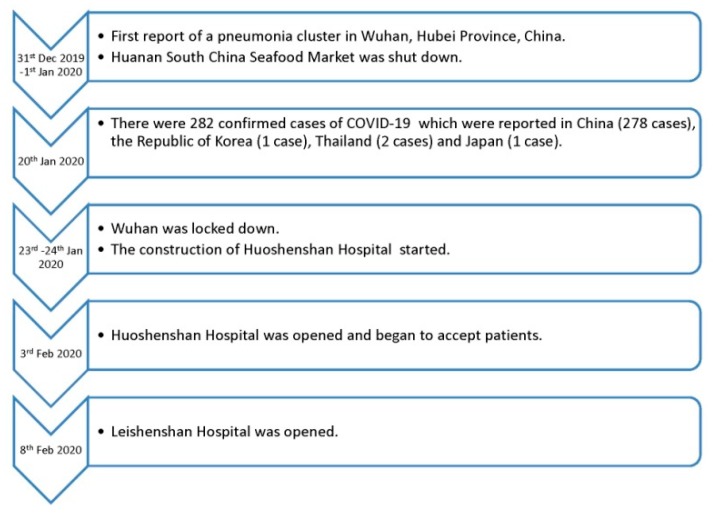
Timeline of the key COVID-19 events in China.

To our knowledge, our study was among one of the first studies to investigate the immediate impact of the COVID-19 pandemic on the mental health and quality of life of the general public in mainland China [13]. Since the pandemic is not over yet and there is a further spread of the pandemic to other countries such as Italy, it is possible that the COVID-19 pandemic will cause excessive panic and anxiety in residents living inside and outside mainland China because of the increasing number of COVID-19 cases worldwide [13]. Moreover, the Chinese authorities had taken measures to lock down Wuhan and the entire Hubei Province during the Chinese Spring Festival to contain the pandemic [6]. However, there was still a massive banquet that hosted thousands of people held over the holiday period (before the city lockdown), during a time when the relevant authorities should have been aware of the severity of the virus and taken appropriate actions to stop such banquets. Therefore, some might argue that the responses from the relevant authorities should have been faster and more proactive. At the same time, two new hospitals (i.e., Huoshenshan Hospital and Leishenshan Hospital) to combat the COVID-19 pandemic were built within only 10 days (Figure 1). However, it was important to make sure that these quarantine facilities were not designed merely for housing large numbers of people, which can, in fact, spread the infection further [2]. Holiday periods were also postponed and school openings were extended to reduce the numbers of new COVID-19 cases [14].

The overall IES score in participants indicated a mild stressful impact. One possible reason for this finding is that the disease outbreak was not regarded as severe during the time that the study was conducted. Additionally, it is possible that participants still might not have been well informed about the severity of the virus, as mentioned previously. At the time when this study was conducted, our city, Jinzhou, Liaoning Province was not locked down as had happened in Wuhan, Hubei Province. Liaoning Province is located in the Northeast of China and the road distance between Liaoning and Hubei Provinces is approximately 1700 km. By March 2020, the number of confirmed COVID-19 cases in Hubei Province is approximately 67,801, which is higher than that of Liaoning Province (i.e., 127 confirmed COVID-19 cases) [3]. Moreover, the majority of participants reported that they received increased social and family support. Our study also documented that most of the participants had positive mental health-related lifestyle changes. Spending more time to rest was also associated with a lower IES score in our participants. Therefore, these factors might have helped to reduce the stressful impact of the COVID-19 pandemic. Future studies should also investigate if limited knowledge, lack of interest, the relationship between the distance of the survey population from the epicenter of the epidemic, or other factors might contribute to such a limited impact on mental health as reported in our study.

Although more than half of the participants (52.1%) reported that they felt horrified and apprehensive due to the COVID-19 pandemic, they did not feel helpless due to the pandemic. Additionally, the majority of participants reported that they were paying more attention to their mental health, spending more time relaxing, resting and exercising after the onset of the pandemic. These positive impacts on mental health may have helped the participants cope with other negative impacts on mental health, including increased stress. Alternatively, an increase in financial and family stress in a disaster could be associated with some avoidance behaviors, which would have worsened their mental health and lead to a more passive lifestyle [15,16].

Our study results were consistent with the findings reported by Lau et al. who investigated mental health and quality of life in Hong Kong residents during the SARS epidemic in 2003 [12]. The authors also reported increased social and family support as well as positive mental health-related lifestyle changes [12]. One possible reason for these findings was that during the pandemic, the pace of the whole society slowed down [12]. This could have then created more opportunities and time among the community members to support and care for each other [12,15,16]. Addition, during the Chinese Spring Festival, family members and friends were much valued and there was increased communication with family members and friends. Family members were more likely to care for each other and spend time together because they were asked to avoid going to public places and stay at home, especially during the Chinese Spring Festival [12,15,16]. Moreover, the Chinese Spring Festival is the most important Chinese festival because it marks the beginning of a new year according to the traditional Chinese calendar. It also signifies an opportunity for a fresh start and a hope of good things to come. Friends were also more likely to send regards to each other via WeChat and/or other social media [12,15,16]. However, Hong Kong residents have far fewer restrictions on their social media use than the Chinese residents currently do.

Our study had several strengths. To the best of our knowledge, our study was one of first studies to offer a unique opportunity to investigate the impact of the COVID-19 pandemic, as this study was conducted less than one week after the lockdown in Wuhan and other cities in Hubei Province was imposed by the Chinese government. This is particularly important, as this study serves as some of the first data about the mental health impacts of the COVID-19 pandemic. Additionally, our study pilot-tested the validity of the questionnaires used to ensure that they were appropriate in our study context and setting. However, our study suffered from the limitations associated with the small number of the sample size, poor adherence to the study and the convenience sampling method, which limited the generalization of our findings to the whole Chinese population in mainland China. It is also possible that the participants were limited to only those who have the financial, emotional, and mental latitude to actually answer these questions, which might have skewed all of the answers towards those expected of a “healthier” population in this context. Additionally, to what degree these study responses might have been affected by perceived monitoring of responses among our participants remain uncertain. Amid this moment of heightened security, especially cybersecurity aimed at playing down the crisis, any kind of critical responses or indication that may belie a sense of things being out of control would be monitored or suppressed. Additionally, the possible recall bias from participants may have confounded our findings. The income level of participants was not assessed in our study. Although we collected the occupational information of the participants, we did not collect specific details including whether their occupation was related to healthcare. Additionally, we did not collect information on whether participants had a relative/friend who contracted the virus or who developed symptoms.

We did not use any promotional material for the survey because during this sensitive time, the extent to which participants may reasonably perceive the survey as a kind of institutional surveillance may have impacted the validity of the responses. Participants were asked to refer to the information regarding COVID-19 updates and the prevention measures released by the government. Large-scale studies with both qualitative and quantitative methods should be conducted in all regions of China to investigate the mental health and quality of life among Chinese residents, especially in the areas most severely impacted by the pandemic (e.g., Wuhan and other cities in Hubei Province). Following the current study, we will conduct a long-term follow-up study on these same participants as well as a large-scale survey to explore whether there were any significant changes in the mental health impact of the COVID-19 pandemic. We will also investigate whether these participants develop post-traumatic stress after the COVID-19 pandemic is over. Hopefully by then, we will have some ideas on what transmits the SARS-CoV-2 virus and how this virus comes through. This will then provide some important information for community health workers in mainland China to help them tackle these mental health-related issues in response to other similar societal disasters. It is also imperative for mental health workers to be aware that such traumatic stress symptoms could lead to the development of avoidance behaviors or passive lifestyles after the pandemic. We strongly urge that health workers should include mental health promotion as part of their follow-up after the pandemic.

Future studies should also incorporate more nuanced research questions. For example, if younger participants express a stronger sense of helplessness, which may be correlated with an increased use of social media. This is because young participants may watch and listen to more and more negative news which will then intensify their feelings of anxiety and depression in times of crisis. Therefore, questions about social media use, or internet use, or news consumption, would be helpful to understand the impact of such epidemics on mental health. Moreover, questions about relatives/friends who have contracted the virus, health history of the individuals, and their relationship with healthcare sectors (for work), and existing mental health issues should also be included.

## 5. Conclusions

The COVID-19 pandemic was associated with mild stressful impact in our sample; since the COVID-19 pandemic is still ongoing, these findings need to be confirmed and investigated in future larger population studies. Our study managed to capture some immediate positive and negative mental health impacts of the COVID-19 pandemic. Our study has also suggested some important future research areas to assess the impact of the COVID-19 pandemic.

## Figures and Tables

**Table 1:** Sociodemographic characteristics of participants.

Variables	All (*n* = 263)	Females (*n* = 157)	Males (*n* = 106)	*P*-Value
Age (years)	37.7 ± 14.0	35.9 ± 14.5	40.3 ± 12.8	0.010
BMI (kg/m^2^)	22.9 ± 4.3	21.8 ± 2.9	24.5 ± 5.3	<0.001
Education level, n (%)				
*Secondary school*	66 (25.1)	39 (24.8)	27 (25.5)	0.908
*Higher qualification*	197 (74.9)	118 (75.2)	79 (74.5)	
Marital status, n (%)				
*Single/Divorced*	103 (39.2)	73 (46.5)	30 (28.3)	0.003
*Married*	160 (60.8)	84 (53.5)	76 (71.7)	
Employment status, n (%)				
*Full-time*	138 (52.5)	75 (47.8)	63 (59.4)	0.018
*Part-time*	42 (16.0)	22 (14.0)	20 (18.9)	
*Students*	83 (31.6)	60 (38.2)	23 (21.7)	
Religion, n (%)				
*No religion*	250 (95.1)	148 (94.3)	102 (96.2)	0.328
*Buddhist*	11 (4.2)	7 (4.5)	4 (3.8)	
*Christian*	2 (0.8)	2(1.3)	0 (0.0)	

**Table 2:** Multiple linear regression analysis ^1^.

Variables	B	Std. Error	Beta	t	*P*-Value
*Constant*	8.12	0.3693	–	2.199	0.029
Age	0.026	0.037	0.048	0.706	0.481
Sex	−1.794	1.019	−0.115	−1.76	0.080
BMI	0.139	0.118	0.077	1.175	0.241
Education	1.185	1.171	0.067	1.013	0.312

^1^ IES score as a dependent continuous variable.

**Table 3:** IES and negative health impacts by demographic factors.

	Sex (*n* = 263)		*P*-Value ^1^	Age Group (Years) (*n* = 263)				*P*-Value ^1^	Education Level (*n* = 263)		*P*-Value ^1^
Variables	Females (*n* = 157)	Males (*n* = 106)		18–30 (*n* = 109)	31–40 (*n* = 46)	41–50 (*n* = 54)	>50 (*n* = 54)		Secondary School (*n* = 66)	Higher Qualification (*n* =197)	
IES	14.2 ± 7.8	12.8 ± 7.4	0.173 ^2^	13.9 ± 8.1	13.1 ± 6.2	12.6 ± 7.0	14.5 ± 8.5	0.583 ^3^	13.0 ± 7.6	13.8 ± 7.7	0.439 ^2^
IES ≥26, n (%)	10 (6.4)	10 (9.4)	0.478	9 (8.3)	1 (2.2)	4 (7.4)	6 (11.1)	0.560	5 (7.6)	15 (7.6)	1.000
Increased stress from work, n (%)											
*Yes*	50 (31.8)	31 (29.2)	0.685	34 (31.2)	16 (34.8)	10 (18.5)	21 (38.9)	0.120	15 (22.7)	66 (33.5)	0.124
*No*	107 (68.2)	75 (70.8)		75 (68.8)	30 (65.2)	44 (81.5)	33 (61.1)		51 (77.3)	131 (66.5)	
Increased financial stress, n (%)											
*Yes*	37 (23.6)	24 (22.6)	0.883	28 (25.7)	10 (21.7)	8 (14.8)	15 (27.8)	0.362	11 (16.7)	50 (25.4)	0.178
*No*	120 (76.4)	82 (77.4)		81 (74.3)	36 (78.3)	46 (85.2)	39 (72.2)		55 (83.3)	147 (74.6)	
Increased stress from home, n (%)											
*Yes*	37 (23.6)	30 (28.3)	0.391	31 (28.4)	12 (26.1)	9 (16.7)	15 (27.8)	0.412	13 (19.7)	54 (27.4)	0.254
*No*	120 (76.4)	76 (71.7)		78 (71.6)	34 (73.9)	45 (83.3)	39 (72.2)		53 (80.3)	143 (72.6)	
Feel horrified due to the COVID-19, n (%)											
*Yes*	83 (52.9)	54 (50.9)	0.802	67 (61.5)	25 (54.3)	16 (29.6)	29 (53.7)	0.002	29 (43.9)	108 (54.8)	0.155
*No*	74 (47.1)	52 (49.1)		42 (38.5)	21 (45.7)	38 (70.4)	25 (46.3)		37 (56.1)	89 (45.2)	
Feel apprehensive due to the COVID-19, n (%)											
*Yes*	83 (52.9)	54 (50.9)	0.802	63 (57.8)	27 (58.7)	15 (27.8)	32 (59.3)	0.001	33 (50.0)	104 (52.8)	0.776
*No*	74 (47.1)	52 (49.1)		46 (42.2)	19 (41.3)	39 (72.2)	22 (40.7)		33 (50.0)	93 (47.2)	
Feel helpless due to the COVID-19, n (%)											
*Yes*	74 (47.1)	49 (46.2)	0.900	56 (51.4)	26 (56.5)	17 (31.5)	24 (44.4)	0.049	32 (48.5)	91 (46.2)	0.777
*No*	83 (52.9)	57 (53.8)		53 (48.6)	20 (43.5)	37 (68.5)	30 (55.6)		34 (51.5)	106 (53.8)	

^1^ Chi-square test or Chi-square test for trend was used for variables except for IES score. ^2^ P-value was based on unpaired *t*-test. ^3^ P-value was based on GLM univariate analysis test.

**Table 4:** Changes in family and social support by demographic factors.

	Sex (*n* = 263)		*P*-Value ^1^	Age Group (Years) (n = 263)				*P*-Value ^1^	Education Level (n = 263)		*P*-Value ^1^
Variables	Females (*n* = 157)	Males (*n* = 106)		18–30 (*n* = 109)	31–40 (*n* = 46)	41–50 (*n* = 54)	>50 (*n* = 54)		Secondary School (*n* = 66)	Higher Qualification (*n* = 197)	
Getting support from friends, n (%)											
*Decreased*	6 (3.8)	11 (10.4)	0.080	2 (1.8)	3 (6.5)	8 (14.8)	4 (7.5)	0.028	4 (6.1)	13 (6.6)	0.709
*Same as before*	44 (28.0)	32 (30.2)		30 (27.5)	11 (23.9)	20 (37.0)	15 (27.8)		18 (27.3)	58 (29.4)	
*Increased*	107 (68.2)	63 (59.5)		77 (70.7)	32 (69.6)	26 (47.2)	35 (64.8)		44 (66.7)	126 (64.0)	
Getting support from family members, n (%)											
*Decreased*	11 (7.0)	14 (13.2)	0.126	3 (2.7)	3 (6.5)	11 (20.4)	8 (14.8)	0.008	8 (12.1)	17 (8.6)	0.442
*Same as before*	39 (24.8)	31 (29.2)		29 (26.6)	11 (23.9)	21 (38.9)	9 (16.7)		20 (30.3)	50 (25.4)	
*Increased*	107 (68.2)	61 (57.5)		77 (70.7)	32 (69.6)	22 (40.7)	37 (68.5)		38 (57.6)	130 (66.0)	
Shared feeling with family members, n (%)											
*Decreased*	18 (11.5)	11 (10.4)	0.563	5 (4.6)	8 (17.4)	8 (14.9)	8 (14.8)	0.027	6 (9.1)	23 (11.7)	0.535
*Same as before*	45 (28.7)	37 (34.9)		38 (34.9)	9 (19.6)	22 (40.7)	13 (24.1)		18 (27.3)	64 (32.5)	
*Increased*	94 (59.8)	58 (57.7)		66 (60.5)	29 (63.1)	24 (44.5)	33 (61.2)		42 (63.6)	110 (55.8)	
Shared feeling with others when in blue, n (%)											
*Decreased*	20 (12.7)	9 (8.5)	0.195	5 (4.6)	6 (13.0)	12 (22.0)	6 (11.2)	0.011	10 (15.2)	19 (9.6)	0.452
*Same as before*	36 (22.9)	34 (32.2)		28 (25.7)	8 (17.4)	16 (29.6)	18 (33.3)		16 (24.2)	54 (27.4)	
*Increased*	101 (64.3)	63 (59.4)		76 (69.8)	32 (69.6)	26 (48.2)	30 (55.5)		40 (60.6)	124 (62.9)	
Caring for family members’ feelings, n (%)											
*Decreased*	5 (3.2)	4 (3.7)	0.542	2 (1.8)	1 (2.2)	3 (5.6)	3 (5.6)	0.016	2 (3.0)	7 (3.6)	0.541
*Same as before*	26 (16.6)	23 (21.7)		18 (16.5)	3 (6.5)	15 (27.8)	13 (24.1)		15 (22.7)	34 (17.3)	
*Increased*	126 (80.3)	79 (84.6)		89 (81.7)	42 (91.3)	36 (66.6)	38 (70.4)		49 (74.2)	156 (79.2)	

^1^ Chi-square test or Chi-square test for trend was used.

**Table 5:** Awareness and lifestyles by demographic factors.

	Sex (*n* = 263)		*P*-Value ^1^	Age Group (Years) (*n* = 263)				*P*-Value ^1^	Education Level (*n* = 263)		*P*-Value ^1^
	Females (*n* = 157)	Males (*n* = 106)		18–30 (*n* = 109)	31–40 (*n* = 46)	41–50 (*n* = 54)	>50 (*n* = 54)		Secondary School (*n* = 66)	Higher Qualification (*n* = 197)	
Pay attention to mental health, n (%)											
*Decreased*	5 (3.2)	5 (4.7)	0.539	1 (0.9)	1 (2.2)	6 (11.1)	2 (3.7)	0.050	4 (6.1)	6 (3.0)	0.286
*Same as before*	44 (28.0)	31 (29.2)		26 (23.9)	12 (26.1)	26 (48.1)	11 (20.4)		20 (30.3)	55 (27.9)	
*Increased*	108 (68.8)	70 (66.0)		82 (75.2)	33 (71.7)	22 (40.7)	41 (75.9)		42 (63.6)	136 (69.0)	
Time spent to rest, n (%)											
*Decreased*	3 (1.9)	4 (3.8)	0.665	2 (1.8)	1 (2.2)	1 (1.9)	3 (5.6)	0.317	1 (1.5)	6 (3.0)	0.628
*Same as before*	56 (35.7)	37 (34.9)		36 (33.0)	13 (28.3)	30 (55.6)	14 (25.9)		23 (34.8)	70 (35.5)	
*Increased*	98 (62.4)	65 (61.3)		71 (65.1)	32 (69.6)	23 (42.6)	37 (68.5)		42 (63.6)	121 (61.4)	
Time spent to relax, n (%)											
*Decreased*	14 (8.9)	8 (7.5)	0.906	4 (3.7)	4 (8.7)	2 (3.7)	12 (22.2)	0.011	8 (12.1)	14 (7.1)	0.305
*Same as before*	42 (26.8)	30 (28.3)		29 (26.6)	12 (26.1)	22 (40.7)	9 (16.7)		20 (30.3)	52 (26.4)	
*Increased*	101 (64.3)	68 (64.2)		76 (69.7)	30 (65.2)	30 (55.6)	33 (61.1)		38 (57.6)	131 (66.5)	
Time spent to exercise, n (%)											
*Decreased*	4 (2.5)	5 (4.7)	0.206	3 (2.8)	1 (2.2)	2 (3.7)	3 (5.6)	0.793	4 (6.1)	5 (2.5)	0.588
*Same as before*	55 (35.0)	42 (39.6)		42 (38.5)	11 (23.9)	30 (55.6)	14 (25.9)		23 (34.8)	74 (37.6)	
*Increased*	98 (62.4)	59 (55.7)		64 (58.7)	34 (73.9)	22 (40.7)	37 (68.5)		39 (59.1)	118 (59.9)	

^1^ Chi-square test.
